# Structure of the human heterodimeric transporter 4F2hc-LAT2 in complex with Anticalin, an alternative binding protein for applications in single-particle cryo-EM

**DOI:** 10.1038/s41598-022-23270-1

**Published:** 2022-10-30

**Authors:** Jean-Marc Jeckelmann, Thomas Lemmin, Martin Schlapschy, Arne Skerra, Dimitrios Fotiadis

**Affiliations:** 1 Institute of Biochemistry and Molecular Medicine, University of Bern, Bern, Switzerland; 2 Swiss National Centre of Competence in Research (NCCR) TransCure, University of Bern, Bern, Switzerland; 3 Lehrstuhl Für Biologische Chemie, Technische Universität München, Freising, Germany

**Keywords:** Cryoelectron microscopy, Carrier proteins, Membrane proteins

## Abstract

Cryo-EM structure determination of relatively small and flexible membrane proteins at high resolution is challenging. Increasing the size and structural features by binding of high affinity proteins to the biomolecular target allows for better particle alignment and may result in structural models of higher resolution and quality. Anticalins are alternative binding proteins to antibodies, which are based on the lipocalin scaffold and show potential for theranostic applications. The human heterodimeric amino acid transporter 4F2hc-LAT2 is a membrane protein complex that mediates transport of certain amino acids and derivatives thereof across the plasma membrane. Here, we present and discuss the cryo-EM structure of human 4F2hc-LAT2 in complex with the anticalin D11vs at 3.2 Å resolution. Relative high local map resolution (2.8–3.0 Å) in the LAT2 substrate binding site together with molecular dynamics simulations indicated the presence of fixed water molecules potentially involved in shaping and stabilizing this region. Finally, the presented work expands the application portfolio of anticalins and widens the toolset of binding proteins to promote high-resolution structure solution by single-particle cryo-EM.

## Introduction

Amino acids are important metabolites involved in various cellular processes ranging from signaling and energy production to protein synthesis. Transport of amino acids across biological membranes is mediated by membrane proteins, which in mammals belong to different solute carrier (SLC) families^1^. One of them is the SLC7 family, which comprises 15 genes^2^ of amino acid transporters from the amino acid, polyamine and organocation (APC) superfamily of secondary carriers (transport classification (TC) system No. 2.A.3; http://www.tcdb.org)^3^. The SLC7 family can be divided into two subgroups, i.e., the cationic amino acid transporters (CATs; SLC7A1-A4 and SLC7A14) and the glycoprotein-associated L-type amino acid transporters (LATs; SLC7A5-A11, Slc7a12, SLC7A13 and Slc7a15)^2^. While CATs are N-glycosylated, LATs are not but they associate with type-II membrane N-glycoproteins from the SLC3 family, i.e., either with 4F2hc (SLC3A2; CD98hc) or with rBAT (SLC3A1), to form heterodimeric amino acid transporters (HATs). These two ancillary proteins are also known as ‘heavy chains’ and are composed of an intracellular N-terminal domain, a single transmembrane α-helix and a large extracellular C-terminal domain. Important features of 4F2hc and rBAT heavy chains are that i) they are covalently linked to corresponding ‘light chains’, i.e., LATs, via a conserved disulfide bridge, and ii) in mammalian cells they act as chaperones for correct membrane trafficking and stabilization of the HAT^2,4–6^. The extracellular domain of the heavy chain 4F2hc shares structural similarities to bacterial glucosidases but is enzymatically inactive^7,8^. Light chains are polytopic membrane proteins with both termini located on the cytoplasmic side, which function as the substrate transporters^2^.

The HAT 4F2hc-LAT2 (SLC3A2-SLC7A8) is a Na^+^-independent obligatory exchanger that preferably transports neutral as well as small L-amino acids^9–12^ and the thyroid hormones T3 and T4^13,14^. LAT2 is ubiquitously expressed in tissues^2^ and has implications in human health. For example, loss of LAT2-dependent substrate transport function is attributed to age-related hearing loss (ARHL)^15^ and cataract formation^16^. Recently, coding variants of LAT2 were linked to an increased risk to develop autism spectrum disorder^17^. Furthermore, elevated expression levels of LAT2 were reported to be associated with basal cell carcinoma and pancreatic cancer^18,19^. Thus, for this types of cancer, 4F2hc-LAT2 may represent a potential pharmacological target and diagnostic marker.

The structure of the heavy chain 4F2hc-ectodomain (4F2hc-ED) was solved by X-ray crystallography^7,20^ and structural information of the 4F2hc-LAT2 heterodimeric complex at different resolutions was acquired by electron microscopy (EM) including cryo-EM^6,21–25^. For structure determination by cryo-EM, detergent-solubilized, purified membrane proteins are embedded in detergent micelles. The resulting unstructured, micellar density may impair with the ability to align particles properly, especially for relatively small and dynamic membrane proteins without internal symmetry such as 4F2hc-LAT2^23^. Increasing the size and structural features as well as reducing protein flexibility of the target protein is an elegant solution to overcome this limitation. To this aim, specific binding proteins towards the protein of interest such as fragment antigen binding (Fab) and camelid heavy-chain-only antibody fragments (nanobodies) have been utilized^26–29^. Larger binding proteins composed of Fabs and nanobodies that are either grafted or bound to scaffold proteins have also been reported recently^30–32^. Anticalins, a class of engineered ligand-binding proteins^33^, appear attractive as a new type of binding protein for single-particle cryo-EM. They are derived from the human lipocalin scaffold and represent a valuable alternative to antibodies and nanobodies^34^. Anticalins are composed of a central eight-stranded antiparallel β-barrel backed by an extended strand and an α-helical segment. The binding region to the target protein is at the open end of the β-barrel and formed by four structurally variable loops^35^. Recently, an anticalin (D11vs) directed against the 4F2hc-ED displaying high affinity in the low picomolar region was reported^36^.

In this study, we explored the potential of anticalins as binding proteins for single-particle cryo-EM structure determination of 4F2hc-dependent HATs. We overexpressed the human HAT 4F2hc-LAT2 in the yeast *Pichia pastoris*, purified the protein in the presence of detergent and solved its structure by cryo-EM at 3.2 Å resolution after complex formation with the anticalin D11vs produced in *E. coli*. In addition, densities corresponding to water molecules that could act as placeholders for ligand atoms in the apo-state structure were identified in the substrate binding pocket and their existence supported by molecular dynamics simulations.

## Results and discussion

### Preparation of the 4F2hc-LAT2::D11vs complex

**Figure 1:**
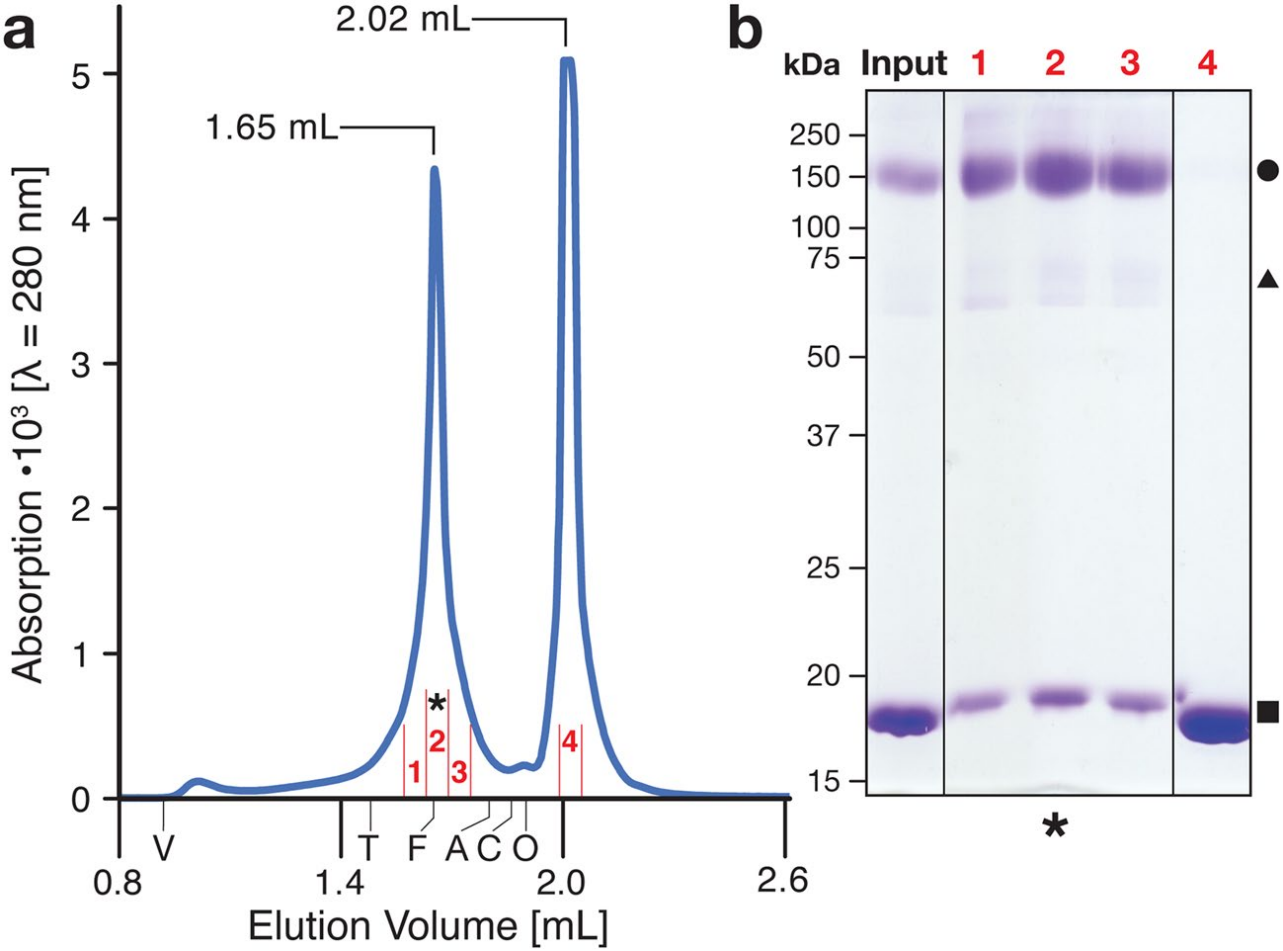
Size-exclusion chromatography (SEC) and SDS-PAGE analysis of the purified human 4F2hc-LAT2::D11vs complex. (**a**) Representative SEC elution profile displaying the separation of 4F2hc-LAT2 complexed to D11vs (elution peak at 1.65 mL) from free D11vs (elution peak at 2.02 mL) using a Superose 6 Increase 3.2/300 column. The void volume (V) of the column and retention volumes of the standard proteins such as thyroglobulin (T, 669 kDa), ferritin (F, 440 kDa), aldolase (A, 158 kDa), conalbumin (C, 75 kDa) and ovalbumin (O, 43 kDa) are indicated. (**b**) SDS-PAGE (10% Coomassie Brilliant Blue stained SDS/polyacrylamide gel, ~ 2 µg of protein loaded) of samples before (Input) and after SEC. Lanes with red numbered labels 1 to 4 correspond to the samples labeled as fractions in panel a. Note that the peak fraction at an elution volume of 1.65 mL is composed of two major (at ~ 150 kDa: 4F2hc-LAT2, filled circle and ~ 18 kDa: D11vs, filled square) and minor (4F2hc, filled triangle) protein bands, whereas the elution peak at 2.02 mL (fraction 4 in panel a) is composed of only one major protein band, i.e. the excess anticalin. Fraction 2 was used for cryo-EM and is additionally labeled with an asterisk. The uncropped SDS-PAGE gel to panel b is provided in Supplementary Fig. S5.

The human HAT 4F2hc-LAT2 was expressed as a recombinant protein in the methylotrophic yeast *P. pastoris* and purified as described previously^37^. Pure and disulfide-linked heterodimeric 4F2hc-LAT2 solubilized in glyco-diosgenin (GDN) was mixed with an excess of the purified anticalin D11vs, which had been produced in the periplasm of *E. coli*^36^. The resulting 4F2hc-LAT2::D11vs complex was isolated by size-exclusion chromatography (SEC) (Fig. 1). SDS–polyacrylamide gel electrophoresis (SDS-PAGE) of the SEC peak fraction at an elution volume of 1.65 mL (Fig. 1a) showed two major protein bands migrating at ~ 150 kDa and ~ 18 kDa, respectively, which correspond to covalently linked 4F2hc-LAT2 and D11vs (labeled with a filled circle and square in Fig. 1b). The minor bands at 60–75 kDa are attributed to glycosylated 4F2hc proteins from HATs that are disrupted by SDS-denaturation and/or during SDS-PAGE^23,24,38^.

### Structure determination and overall structure the 4F2hc-LAT2::D11vs complex

**Figure 2:**
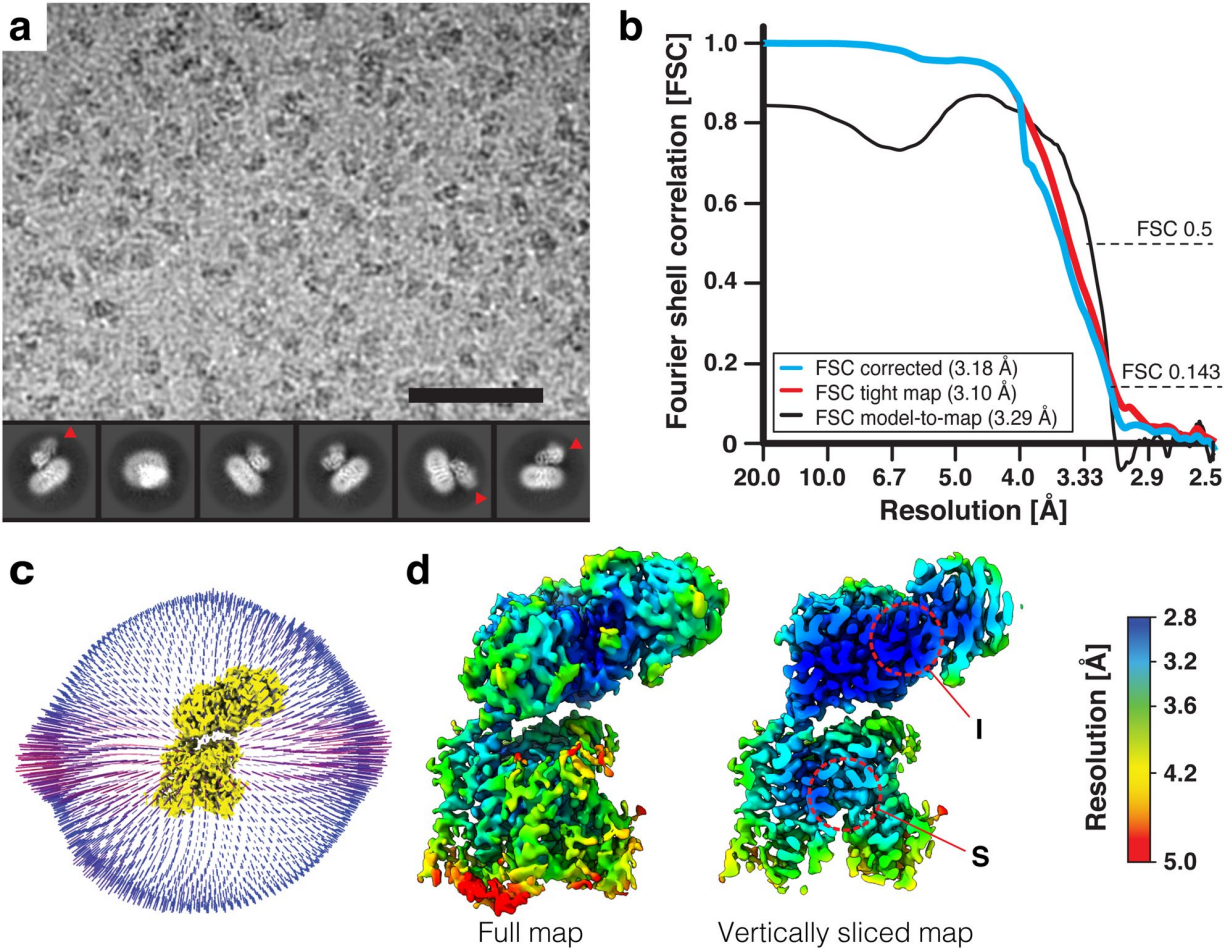
Cryo-EM structure determination of human 4F2hc-LAT2::D11vs. (**a**) In the upper part, a representative electron micrograph recorded at a defocus of about -1.8 μm is shown (scale bar: 50 nm). In the lower part, six selected 2D-class averages are displayed (frame size: 21 nm). Densities of D11vs in 2D-class averages of complexes viewed from the side are indicated with red arrowheads. (**b**) Plot of Fourier shell correlation (FSC) curves, experimental half-maps (FSC = 0.143 criterion) and model-to-map fit (FSC = 0.5 criterion). (**c**) Three-dimensional representation of the Euler angular distribution. The height of blue to red cylinders indicate relative representations of particle orientations. In the center of the plot, the 4F2hc-LAT2::D11vs cryo-EM map is displayed as yellow colored surface. (**d**) Full density map (left) and a vertically sliced map version (right) colored by local resolution and shown at a threshold level of 0.142. Highest resolution regions corresponding to the substrate binding pocket of LAT2 and the interface between 4F2hc-ED and D11vs are labeled with S and I, respectively. Volume representations were prepared using UCSF ChimeraX (version 1.3; see Materials and Methods). The figure was assembled and labeled using Adobe Illustrator 26.5 (https://adobe.com/products/illustrator).

The peak fraction of the protein-complex (Fig. 1a, labeled with an asterisk) was used for the cryo-electron microscopic studies. Data acquisition and processing information is provided in the Materials and Methods section as well as in Supplementary Fig. S1 and Supplementary Table S1. Cryo-EM images displayed a homogenous distribution of the GDN-solubilized 4F2hc-LAT2::D11vs complex, and corresponding 2D class averages showed the typical bilobed 4F2hc-LAT2 transporter structure (Fig. 2a)^6,21–25^. Compared to side view 2D class averages of heterodimeric 4F2hc-LAT2 particles^23^, a characteristic additional density at the 4F2hc-ED was observed and attributed to the anticalin D11vs (Fig. 2a, red arrowheads). The overall resolution of the final cryo-EM 3D density map calculated from differently oriented 4F2hc-LAT2::D11vs complex particles was estimated to 3.2 Å according to the gold standard Fourier shell correlation (FSC) cut-off criterion of 0.143^39,40^ (Fig. 2b,c). Local resolution analysis of the map indicated that the highest resolution features (2.8–3.0 Å) were located in the substrate binding region of LAT2 (S) and in the interface region between 4F2hc-ED and D11vs (I) (labels S and I in Fig. 2d).

**Figure 3:**
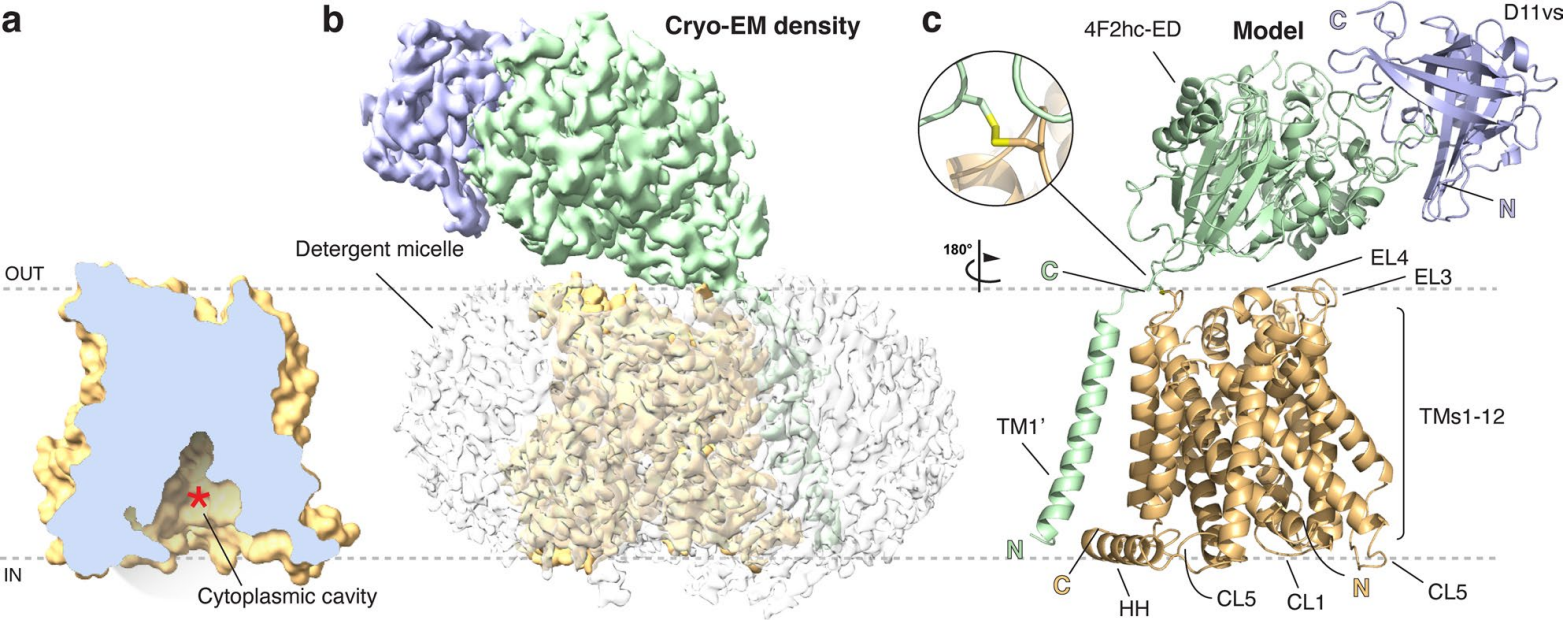
Overall structure of the 4F2hc-LAT2::D11vs complex. (**a**) Displayed is a vertical slice through the LAT2 structure represented as surface to highlight the large cytoplasmic cavity (red asterisk), reflecting an inward-open structure. (**b**) Representation of the cryo-EM density including the detergent micelle (transparent density). (**c**) Structural model of the complex. The magnification reflects the region of the disulfide bridge between 4F2hc (C210) and LAT2 (C154). Structural elements such as transmembrane α-helices (TM), extracellular (EL) and cytoplasmic loops (CL), and the horizontal α-helix (HH) are labeled. 4F2hc, LAT2 and D11vs are colored in green, orange and violet, respectively. Dotted lines represent the membrane boundaries facing the extracellular (OUT) and cytoplasmic (IN) environments. All volume and structural representations were prepared using UCSF ChimeraX (version 1.3) and PyMol (version v2.5.2; The PyMol Molecular Graphics System, Schrödinger), see Materials and Methods. The figure was assembled and labeled using Adobe Illustrator 26.5 (https://adobe.com/products/illustrator).

The complete structure of the 4F2hc-LAT2 heterodimer in complex with the anticalin D11vs was modeled into the cryo-EM density map, except for the N- and C-terminal regions of the individual proteins, where no density was visible. The structure of 4F2hc-LAT2 with the bound anticalin D11vs was captured in the inward-open conformation as evidenced by the large cavity (Fig. 3a, red asterisk) facing the cytoplasm. In addition to clear and continuous protein density, unstructured density surrounding transmembrane α-helical segments of 4F2hc and LAT2 was found and attributed to the GDN-micelle (Fig. 3b). LAT2 is covalently linked to 4F2hc via a disulfide bridge (Fig. 3c). The 4F2hc-ED sits on top of LAT2 and forms a complex with D11vs (Fig. 3c). The cryo-EM density of the type-II membrane N-glycoprotein 4F2hc with its N- and C-termini located on the cytoplasmic- and the extracellular side, respectively, was of high quality, and the structure could be modeled from residue G177, located in the cytoplasm, followed by the transmembrane α-helix of 4F2hc (TM1’) up to the C-terminal end, residue A630, which is located extracellularly. The N-terminal region of 4F2hc remained experimentally unresolved, which can most likely be attributed to the intrinsic flexibility of this unstructured region as predicted by Alphafold (Structure-ID: AF-P08195-F1^41,42^). The visible LAT2 density allowed all structural elements including the 12 transmembrane α-helical segments (TMs) to be built (Fig. 3c and Supplementary Fig. S2). The high local resolution and quality of the cryo-EM density permitted modelling of D11vs with high confidence (Figs. 2d, 3). The anticalin D11vs is a double mutant (F71S, G81V) of the parent version P3D11, and exhibits both, better thermostability and higher affinity towards 4F2hc-ED^36^. When compared to the X-ray crystallographic structure of P3D11^36^, no alteration in the anticalin main chain structure at positions F71S and G81V was observed. Side chain densities in the cryo-EM structure showed unambiguously that F71 and G81 had been replaced by serine (S71) and valine (V81), thus confirming the sequence of D11vs (Supplementary Fig. S3a). The resolution of the cryo-EM density was above 3.6 Å only in the region of 4F2hc and LAT2 that faces the cytoplasm (Fig. 2d). This is reflected by an increase in the mean atomic B-factor values from 53 to 103 Å^2^ when compared to the rest of the complex. On the other hand, the best resolved structural parts, with estimated local resolutions as high as ~ 2.8 Å, are found in the regions of the interface between 4F2hc-ED and the anticalin D11vs and at the center of LAT2, which represents the substrate binding domain (Fig. 2d).

### Comparison of 4F2hc-ectodomain structures

The structure of the human 4F2hc-ED was previously solved by X-ray crystallography, either alone^7^ or in complex with the anticalin P3D11^36^. The human 4F2hc-ED model obtained here is similar to the structures previously reported (Table 1). The alignment of 4F2hc-EDs from the complex structures with anticalins P3D11 (X-ray) and D11vs (cryo-EM) also shows that the protein binding mode is retained (Supplementary Fig. S3b). The largest Cα displacement is about 5 Å and found at residue H236, which is in a highly flexible glycine-rich loop of the 4F2hc-ED (Supplementary Fig. S3c).

**Table 1:** Comparison between the structure described here and previously published 4F2hc-ED and 4F2hc-LAT2 structures.

Bound ligand	PDB-ID	Reported resolution [Å]	Structural alignment to our structure [r.m.s.d X in Å/for Y Cα-atoms]^a^		Remarks (Reference)
			4F2hc only	LAT2 only	
None	2DH2	2.10	0.423/364	–	X-ray crystallographic structure of 4F2hc-ED^7^
None	6S8V	1.80	0.537/369	–	X-ray crystallographic structure of 4F2hc-ED::P3D11^36^
None	7B00	3.98	0.878/426	0.721/375	Cryo-EM structure of 4F2hc-LAT2^22^
L-Leu	7CMI	2.90	0.644/413	0.701/374	Cryo-EM structure of 4F2hc-LAT2^21^
L-Trp	7CMH	3.40	0.638/406	0.651/365	Cryo-EM structure of 4F2hc-LAT2^21^

^a^Alignments were calculated using the command "align” in PyMOL version v2.5.2 (The PyMol Molecular Graphics System, Schrödinger).

### Comparison of LAT2 substrate binding pockets from cryo-EM structures

**Figure 4:**
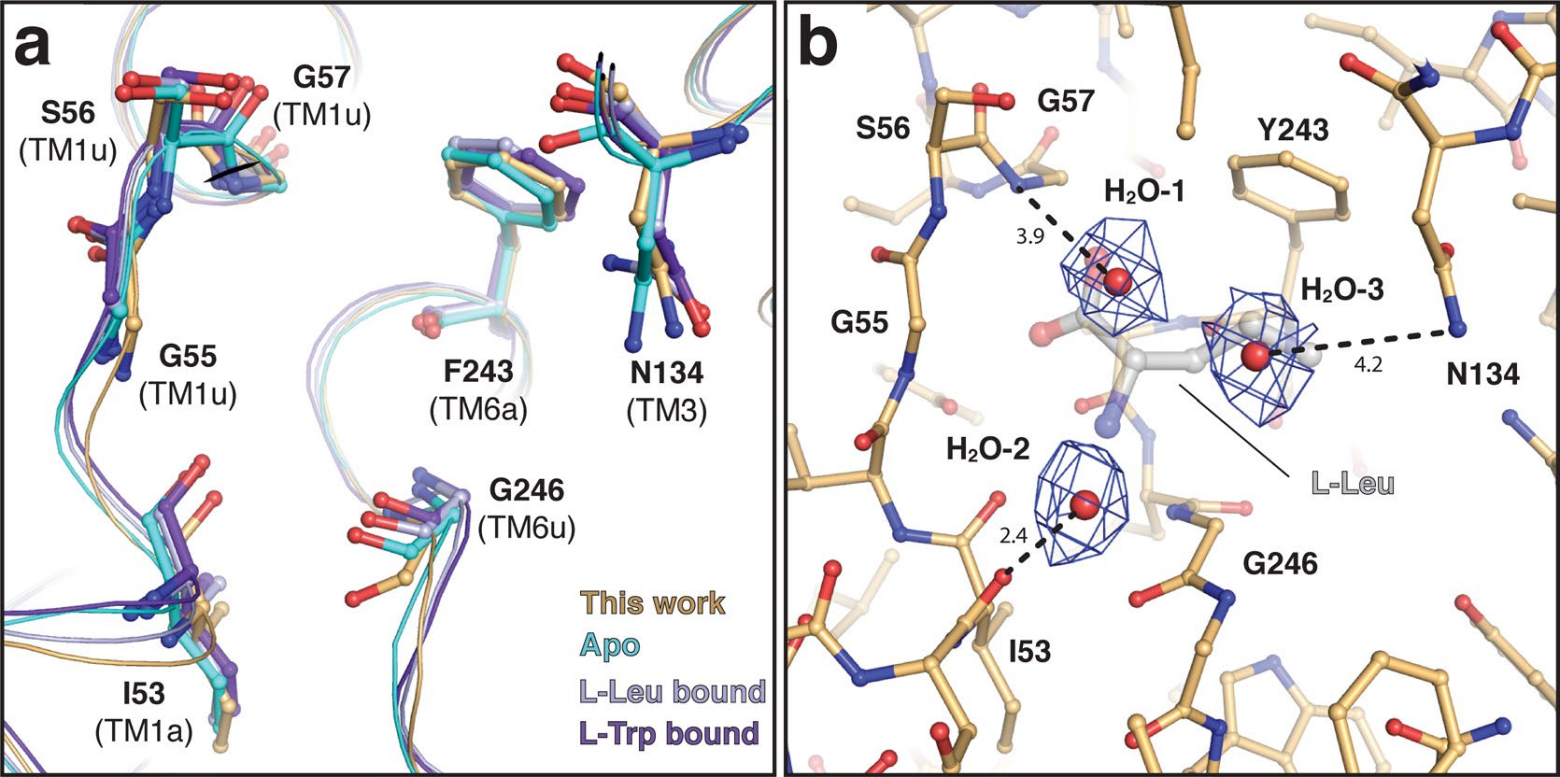
View into the substrate binding pocket of superimposed LAT2 structures and identified water molecules. (**a**) Ribbon representation (thin lines), and residues important for substrate recognition and binding (sticks) for the following 4F2hc-LAT2 heterodimer structures: apo (orange; this work), apo (cyan, PDB-ID: 7B00), L-Leu bound (light blue, PDB-ID: 7CMI) and L-Trp bound (violet, PDB-ID: 7CMH). (**b**) Substrate binding pocket of 4F2hc-LAT2::D11vs (this work) including the identified water molecules H_2_O-1, H_2_O-2 and H_2_O-3, and their respective cryo-EM densities. Displayed is also the substrate L-Leu (faint grey sticks) from the superimposed L-Leu bound 4F2hc-LAT2 structure (Supplementary Fig. S4b). Structural representations were prepared using PyMol (version v2.5.2; The PyMol Molecular Graphics System, Schrödinger). The figure was assembled and labeled using Adobe Illustrator 26.5 (https://adobe.com/products/illustrator).

Currently, three structural models of human 4F2hc-LAT2 have been reported. All LAT2 transporters were captured in the inward-open conformation and either in the apo-state^22^ or with a bound L-amino acid, i.e., L-leucine and L-tryptophan^21^. Individual alignment calculations of the reported LAT2 structures with ours revealed mean Cα displacements of ≤ 0.721 Å (Table 1). Thus, variations in cellular overexpression systems, purification strategies and cryo-EM grid preparations did not affect the structures of LAT2 significantly^21,22^. Residues in the substrate binding pocket, which are involved in hydrogen-bond (H-bond) formation or van der Waals interactions, are located in TM1, TM3 and TM6^21,22^. TM1 and TM6 exhibit central unwound regions (TM1u and TM6u), which connect the α-helical parts TM1a with TM1b and TM6a with TM6b, respectively (Supplementary Fig. S2). A close look into the substrate binding pocket revealed a preserved architecture of protein main chain conformations (Fig. 4a). In addition, only minor side chain displacements were observed for the residues involved in substrate recognition and binding, i.e., I53 (TM1a, H-bonding), the G55-S56-G57-motif (TM1u, H-bonding), N134 (TM3, H-bonding), G246 (TM6u, H-bonding), and the gating residue F243 (TM6a, hydrophobic interaction) (Fig. 4a and Supplementary Fig. S4). Interestingly, three defined densities were found in the substrate binding pocket, which we attributed to water molecules: H_2_O-1, H_2_O-2 and H_2_O-3 (Fig. 4b). Recently, an extensive water network was observed inside the substrate binding pocket of the L-arginine/agmatine transporter AdiC, a bacterial homologue of LAT transporters^43^. Several water molecules acted as placeholders for polar functional groups of substrates. In our structure, H_2_O-1 is 3.9 Å apart from the amide nitrogen atom of G57 (Fig. 4b) and could thus correspond to the location of one oxygen atom of the carboxyl group in an amino acid substrate (Supplementary Fig. S4a). The water molecule H_2_O-2 is located 2.4 Å from the carbonyl oxygen of I53 (Fig. 4b). H_2_O-3 is found 4.2 Å apart from the amide nitrogen of N134 (Fig. 4b), which, in addition to its H-bonding interaction with substrates, was recently suggested to be a key residue responsible for distinct substrate selectivities between human LAT2 and human LAT1 concerning small neutral amino acids and L-glutamine^22^. In order to support the existence of the three observed water molecules in the substrate binding pocket of LAT2, a set of four all-atom molecular dynamics (MD) simulations of the 4F2hc-LAT2::D11vs complex was performed under near physiological conditions. The persistent presence of water molecules at all three positions was confirmed with occupancies of H_2_O-1: 59.3 ± 4.3%, H_2_O-2: 65.1 ± 4.5% and H_2_O-3: 52.7 ± 11.4%.

## Conclusion

Cryo-EM structure solution of relatively small membrane proteins with no internal symmetry, such as 4F2hc-LAT2, generally suffers from suboptimal particle alignment, which renders high-resolution structure determination difficult or even impossible^23^. To overcome this hurdle and provide more volume and structural features for optimal particle alignment, the protein of interest may be complexed with a specific high affinity binding protein^26,30–32^. Anticalins are artificial proteins derived from the human lipocalin scaffold, which are increasingly used in biomedical research as valid alternatives to antibodies for theranostic purposes^44^. Anticalins can be selected from naive combinatorial libraries against a wide range of target molecules including proteins, peptides as well as chemical compounds^34,45^, using routine procedures such as phage display and/or bacterial surface display, followed by ELISA screening^46^. In the case of membrane proteins, anticalins are most easily selected against soluble versions of the extracellular region as previously demonstrated for CTLA-4^47^, PSMA^48^, VEGFR-3^49^ or CD98hc^36^, for example. In cases where the native membrane protein can be overexpressed and solubilized, such preparations can be directly applied for anticalin selection, as demonstrated for the 5-HT_3_ serotonin receptor (M. Trumpfheller, C. Mendler, H. Nury, H. Vogel & A. Skerra, unpublished). Here, we present the structure solution to 3.2 Å resolution of the human heterodimer 4F2hc-LAT2 in complex with the anticalin D11vs. In a previous study, we overexpressed and purified the human 4F2hc-LAT2 heterodimer in the same manner as described here and obtained a cryo-EM density of the human 4F2hc-LAT2 heterodimer at a resolution of ~ 7.5 Å^23^. Thus, complexation of 4F2hc-LAT2 with D11vs^36^ led to a dramatic increase in resolution, which allowed for confident model building (Supplementary Table S1). No significant structural differences were observed between our structure and previously reported 4F2hc-ED and 4F2hc-LAT2 structures (Table 1, Fig. 4, Supplementary Fig. S3b, Supplementary Fig. S4). This indicates that the complexation of 4F2hc-LAT2 with an anticalin did not alter the structure of the heterodimer. Similar to the recently reported structure of the bacterial LAT-homologue AdiC^43^, we found water molecules inside the substrate binding pocket (Fig. 4b), which are potentially involved in shaping and stabilizing this region. In summary, the cryo-EM structure of the 4F2hc-LAT2::D11vs complex at 3.2 Å resolution demonstrated that anticalins represent powerful binding proteins to solve cryo-EM structures of HATs, and potentially other small membrane proteins, at high resolution.

## Materials and methods

### Production and purification of human 4F2hc-LAT2 in complex with D11vs

To heterologously overexpress human 4F2hc-LAT2, a previously characterized and reported *P. pastoris* clone was used^12,14,37^. The recombinant protein was produced in *P. pastoris* as described^23^, solubilized with GDN and purified according to published protocols^37,38^. The anticalin D11vs was produced at preparative scale via secretion in *E. coli* KS272 with a C-terminal Strep-tag II using the plasmid pNGAL98-D11vs^36^. After periplasmic protein extraction, the recombinant protein was purified using a Strep-Tactin Sepharose column (IBA, Göttingen, Germany) and finally subjected to SEC in PBS (4 mM KH_2_PO_4_, 160 mM Na_2_HPO_4_, 115 mM NaCl pH 7.4) on a HiLoad 26/600 Superdex 75 pg column (Cytiva, Freiburg, Germany). The GDN-solubilized 4F2hc-LAT2 heterodimer was mixed with 4 molar equivalents of D11vs and incubated on ice for 1 h followed by concentration to ~ 10 mg/mL with an Amicon 100-kDa molecular weight cut-off device (Merck, Switzerland). This sample was further purified by SEC using a Superose 6 Increase 3.2/300 column (Cytiva, Switzerland) with 20 mM Bis–Tris propane pH 8.0, 150 mM NaCl, 1% (v/v) glycerol, 0.02% (w/v) GDN as running buffer. Individual fractions were analyzed by SDS-PAGE and used for cryo-EM grid preparation.

### Grid preparation and cryo-EM data collection

The SEC peak fraction containing the purified 4F2hc-LAT2::D11vs complex (Fig. 1a) was concentrated to ~ 3 mg/ml using an Amicon 100-kDa molecular weight cut-off device and centrifuged (20,000 × g, 4 °C, 5 min). A protein sample of 3 µL was applied to a R2/1 200 mesh copper holey-carbon grid (Quantifoil, Germany) that has been glow discharged for 25 s at 10 mA and 0.25 mbar. Samples were blotted for 3 s and vitrified in liquid ethane using a Vitrobot Mark IV system (ThermoFisher) at 4 °C and 100% humidity. Data were acquired on a Titan Krios G3 cryo-TEM (ThermoFisher) operated at 300 kV and equipped with a Quantum-K3 direct electron detector (Gatan). A total of 20,512 movies were collected in counting mode at a defocus range of -0.8 to -1.8 μm and a magnification of 105,000x, which corresponds to a calibrated pixel size of 0.822 Å. Movies were recorded for 1.491 s with a dose rate of 1.255 e^−^/Å^2^/frame, resulting in a total accumulated dose on the specimen level of approximately 50.2 e^−^/Å^2^ per exposure (Supplementary Table S1).

### Data processing and model building

A graphical overview of the single particle cryo-EM image processing workflow is summarized in Supplementary Fig. S1. Dose weighting and motion correction of dose-fractionated and gain-corrected movies were performed using MotionCor2 (version 1.4.0)^50^. Parameters for the contrast transfer function (CTF) were estimated using ctffind (version 4.1.14 47)^51^. Images of low quality displaying strong drift and a maximum CTF resolution worse than 6 Å as well as astigmatism greater than 600 Å were excluded from further processing. Initial particle-picking was done using the Laplacian-of-Gaussian-filter-based-picking procedure of Relion (version 3.1.1)^52,53^. 2D-classification templates were created and template-based particle picking was performed in Relion. About 8.2 million of two-fold binned particles were extracted and then imported into cryoSPARC (version 3.3.1)^54,55^. The particles were 2D-classified and particles of selected classes used for an *ab-initio* reconstruction run followed by iterative heterogeneous and non-uniform refinement runs. Of the best 184,000 particles, a star-file was generated using PyEM^56^ to re-extract them without binning in Relion. These particles were again imported into cryoSPARC, 2D-classified and the best 2D-classes used for template-based particle picking on 20,254 patch CTF estimated micrographs. About 4.3 million particles were extracted and the best particles selected by *ab-initio* reconstruction runs followed by iterative heterogeneous- and non-uniform refinement runs that led to a map of about 3.6 Å resolution. A final local refinement using 552,926 particles applying a soft mask to gradually exclude the detergent-micelle yielded a cryo-EM density at 3.18 Å resolution according to the gold standard Fourier shell correlation (FSC) cut-off criterion of 0.143. The option of CTF refinement using cryoSPARC was employed, but no further gain in resolution could be observed. For representation of the Euler angular distribution (Fig. 2c), the cryoSPARC ~.cs-file was converted to a ~.star file and the program “star2bild.py” implemented in PyEM^56^ was applied to generate a ~.bild file. Calculation of the local resolution distribution (Fig. 2d) was done with cryoSPARC.

The coordinates of human 4F2hc (AlphaFold-ID: AF-P08195-F1) and human LAT2 (AlphaFold-ID: AF-Q9UHI5-F1-model_v2)^41,42^, and of the anticalin P3D11 (chain A of PDB-ID: 6S8V) were rudimentarily fitted into the final cryo-EM density using UCSF ChimeraX (version 1.3)^57^. The sequences of 4F2hc and LAT2 were numbered to match UniProt-ID P08195-1 and Q9UHI5-1, respectively, and the sequence of the anticalin P3D11 was mutated to correspond to the D11vs sequence^36^ using Coot^58^. The final model of the 4F2hc-LAT2::D11vs protein complex was obtained by several iterations of real-space refinement using Phenix^59^ and manual model building with Coot^58^, and the structure was validated using MolProbity^60^. All volume and structural representations were prepared using UCSF ChimeraX (version 1.3)^57^ or PyMol (version v2.5.2; The PyMol Molecular Graphics System, Schrödinger).

### Molecular dynamics simulations

The 4F2hc-LAT2::D11 complex was embedded into an 120 × 120 Å plasma membrane mimetic composed of a mixture of palmitoyl-oleoly phosphatidylcholine (POPC) and cholesterol with a ratio of 4:1. The system was then solvated by adding a 17 Å padding layer of water on both sides of the membrane and neutralized with 150 mM NaCl. This system was assembled using the CHARMM-GUI webserver^61^. Finally, 4F2hc was fully glycosylated using Glycosylator^62^.

The simulation was performed with the CHARMM36m force field, including CMAP corrections for the protein^63^. The water molecules were described using the TIP3P parameterization^64^. The simulations were carried out with OPENMM molecular engine^65^, following minimization and equilibration protocols provided by CHARMM-GUI. The cutoff for non-bonded interactions was set to 12 Å with a switching distance at 10 Å. The periodic electrostatic interactions were computed using particle-mesh Ewald (PME) summation with a grid spacing smaller than 1 Å. Constant temperature of 310 K was imposed by Langevin dynamics with a damping coefficient of 1.0 ps. Constant pressure of 1 atm was maintained with Monte Carlo barostat^66^. The hydrogen mass repartitioning scheme was used to achieve a 4 fs time-step^67^.

The simulations were carried out in four replicas up to 200 ns. The trajectories were analysed with VMD^68^ and an in-house script implemented in tcl. The occupancies of H_2_O-1, H_2_O-2 and H_2_O-3 were estimated by measuring the presence of water molecules forming hydrogen bonds with the side chain of N134, or the backbone of S56 or I53, respectively.

## Supplementary Information

Supplementary Information.

## Data Availability

The cryo-EM density map and the protein coordinates were deposited in the Electron Microscopy Data Bank (https://www.ebi.ac.uk/emdb/; EMDB accession code: EMD-15210) and the Protein Data Bank (https://www.rcsb.org; PDB accession code: 8A6L). The uncropped SDS-PAGE gel generated in this study is provided in Supplementary Fig. S5.
