# Supplementary material for: Structure of the human heterodimeric transporter 4F2hc-LAT2 in complex with Anticalin, an alternative binding protein for applications in single-particle cryo-EM
Source: Sci Rep. 2022 Oct 30;12:18269. doi: 10.1038/s41598-022-23270-1 (PMC9618567; doi:10.1038/s41598-022-23270-1)
Supplement: Supplementary file 1 — Supplementary Information. [file 41598_2022_23270_MOESM1_ESM.docx]

Supplementary Information

to

Structure of the human heterodimeric transporter 4F2hc-LAT2 in complex with Anticalin, an alternative binding protein for applications in single-particle cryo-EM

**Jean-Marc Jeckelmann^1,2^, Thomas Lemmin^1^, Martin Schlapschy^3^, Arne Skerra^3^ and Dimitrios Fotiadis^1,2^**

^1^ Institute of Biochemistry and Molecular Medicine, and ^2^ Swiss National Centre of Competence in Research (NCCR) TransCure, University of Bern, Bern, Switzerland

^3^ Lehrstuhl für Biologische Chemie, Technische Universität München, Freising, Germany

**
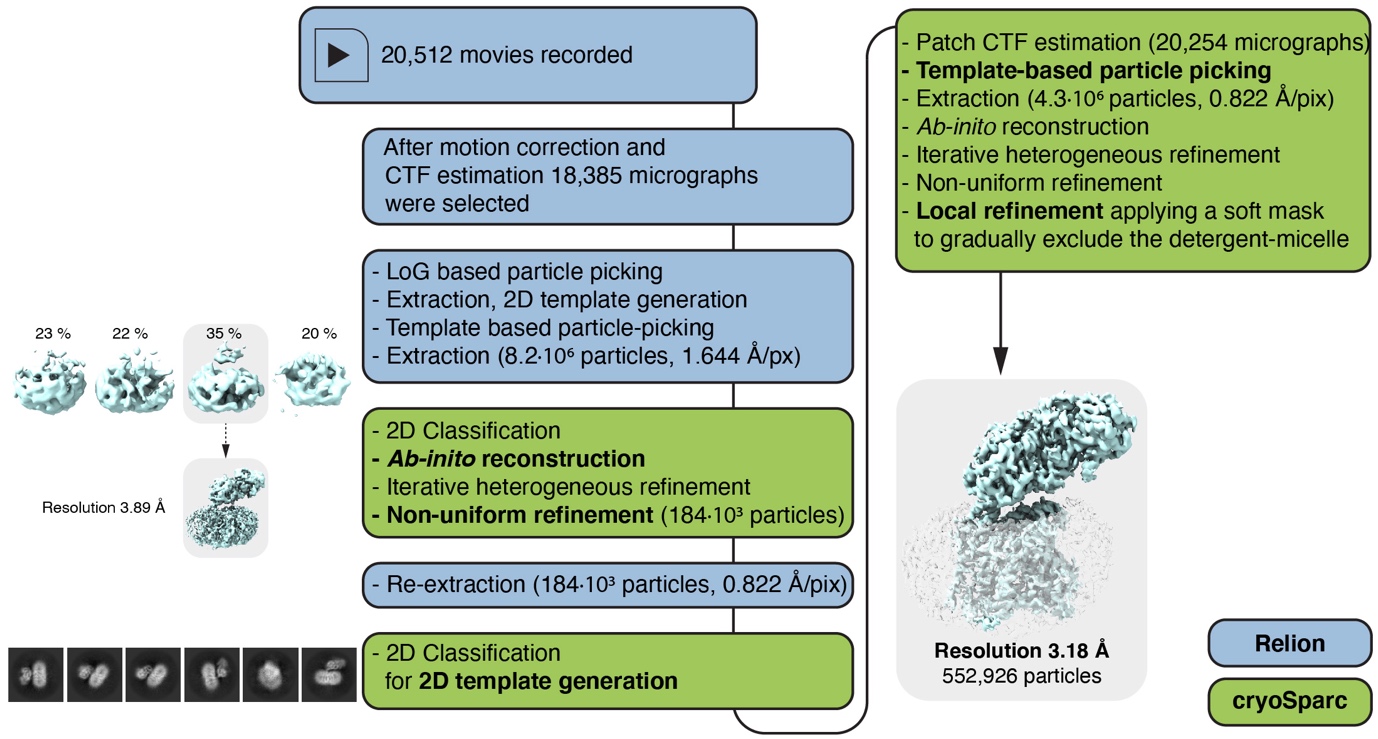
**

**Supplementary Figure S1.** Cryo-EM data processing flow chart. A graphical representation of the processing steps as described in the Materials and Methods section is shown. Calculations were performed using either Relion ^1,2^ or cryoSPARC ^3,4^ (corresponding software highlighted in light blue and green). Displayed cryo-EM 3D maps and 2D-class averages refer to individual steps during data processing and are written in bold face-type. Abbreviations are: CTF, contrast transfer function; LoG, Laplacian-of-Gaussian filter. Volume representations were prepared using UCSF ChimeraX (version 1.3, see Materials and Methods). The figure was assembled and labeled using Adobe Illustrator 26.5 (<https://adobe.com/products/illustrator>).


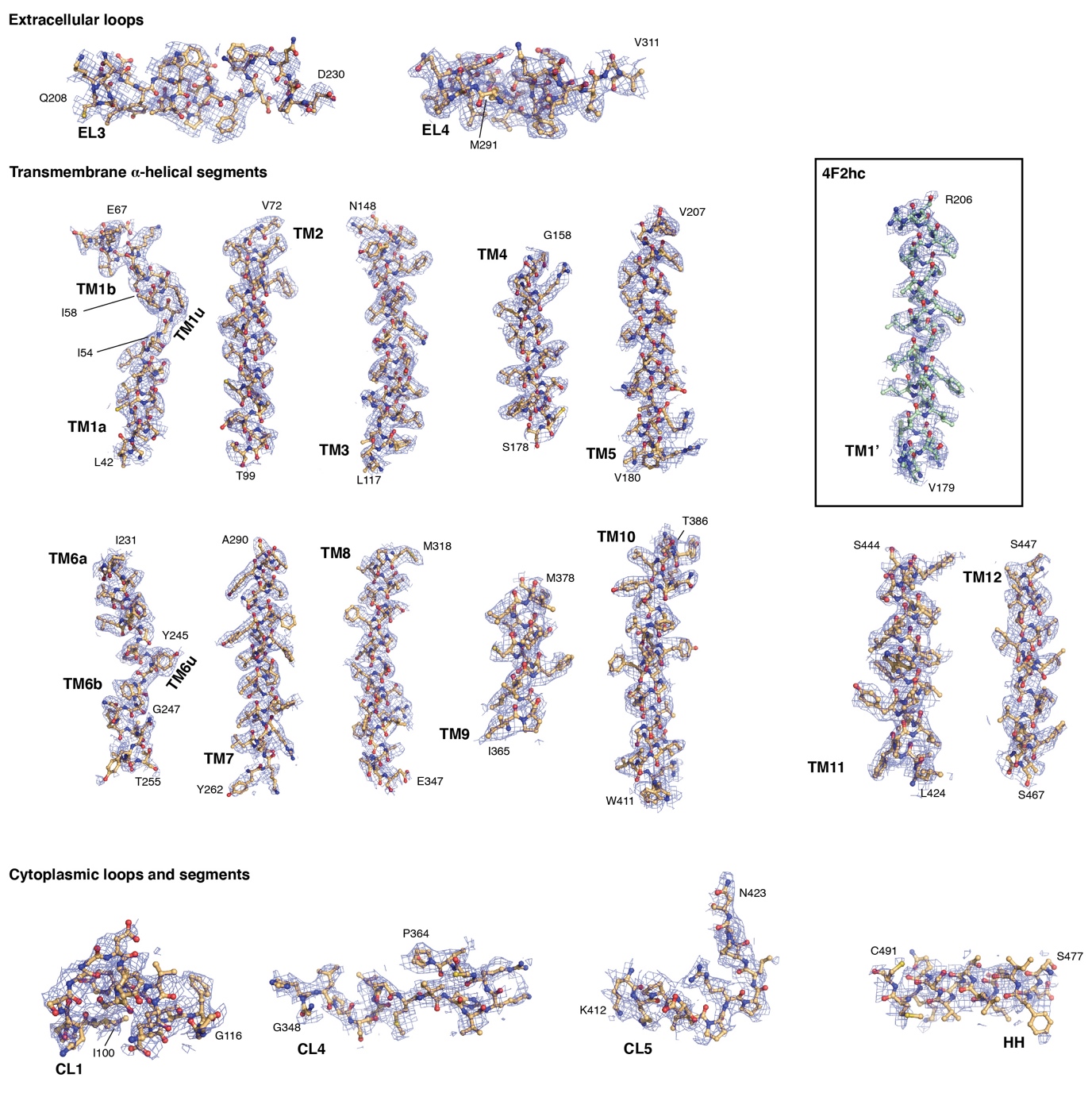


**Supplementary Figure S2.** Cryo-EM density of 4F2hc-LAT2 structural elements. Elements are displayed as sticks (TM1’ of 4F2hc, carbon atoms green; LAT2 elements, carbon atoms orange) and the cryo-EM density as mesh (blue) at a threshold level of 0.125. Shown are all elements indicated in Figure 3c of the main manuscript: TM, transmembrane α-helical segment; EL, extracellular loop; CL, cytoplasmic loop; HH, horizonal α-helix. N- and C-terminal residues are labeled, and the structural element labels are given N-terminally in bold face type. Volume and structural representations were prepared using PyMol (version v2.5.2; The PyMol Molecular Graphics System, Schrödinger). The figure was assembled and labeled using Adobe Illustrator 26.5 (<https://adobe.com/products/illustrator>).


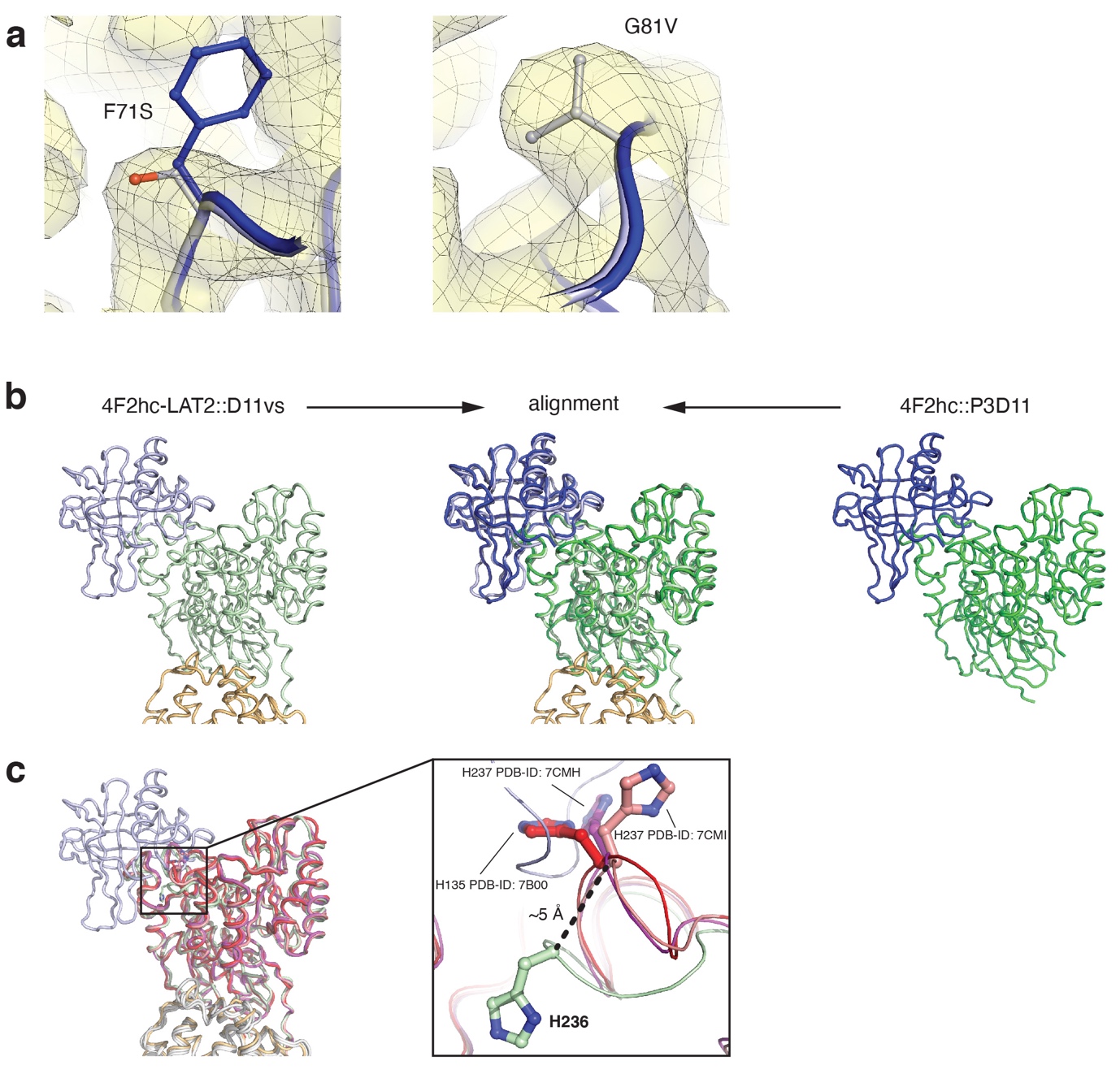


**Supplementary Figure S3.** Anticalin binding to the human 4F2hc ectodomain (4F2hc-ED). (**a**) Displayed is the cryo-EM density at the locations of the point mutations from P3D11 to D11vs, F71S (*left*) and G81V (*right*). (**b**) 4F2hc-ED based structural superposition of 4F2hc-ED::P3D11 (chain B of PDB-ID: 6S8V) onto 4F2hc-LAT2::D11vs (this work). (**c**) 4F2hc-based structural superposition of published 4F2hc-LAT2 cryo-EM structures [PDB-ID: 7B00 (red); 7CMI (pink) and 7CMH (magenta)] onto 4F2hc-LAT2::D11vs (this work). Highlighted on the *right* is the ~5 Å Cα-atomic displacement of H236 upon complexation of 4F2hc with D11vs. R.m.s.d values of the alignments are shown in Table 1 of the main manuscript. Structural representations were prepared using PyMol (version v2.5.2; The PyMol Molecular Graphics System, Schrödinger). The figure was assembled and labeled using Adobe Illustrator 26.5 (<https://adobe.com/products/illustrator>).


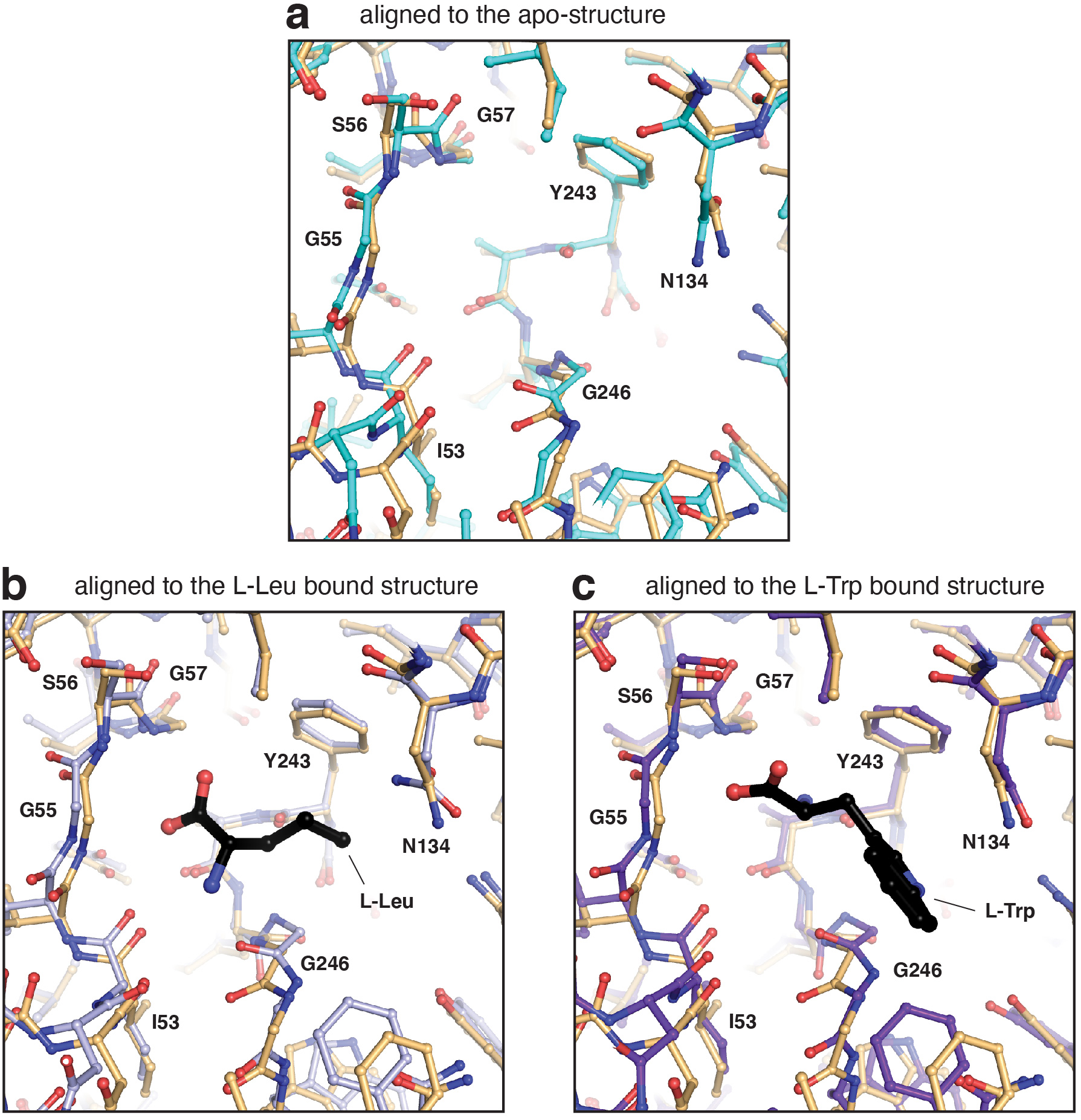


**Supplementary Figure S4.** Detailed view into the substrate binding pocket of human 4F2hc-LAT2. Structural alignments of 4F2hc-LAT2::D11vs (orange, this work) with (**a**) the apo-structure (cyan, PDB-ID: 7B00), (**b**) the L-Leu bound structure (light blue, PDB-ID: 7CMI) and (**c**) the L-Trp bound structure (violet, PDB-ID: 7CMH). Compared to Fig. 4 of the main manuscript, a more detailed structural view is shown by applying a sticks-only representation. Amino acid residues are labeled in the one-letter code. The bound substrates L-Leu (panel b) and L-Trp (panel c) are labeled in the three-letter code and highlighted as black sticks. Structural representations were prepared using PyMol (version v2.5.2; The PyMol Molecular Graphics System, Schrödinger). The figure was assembled and labeled using Adobe Illustrator 26.5 (<https://adobe.com/products/illustrator>).

**
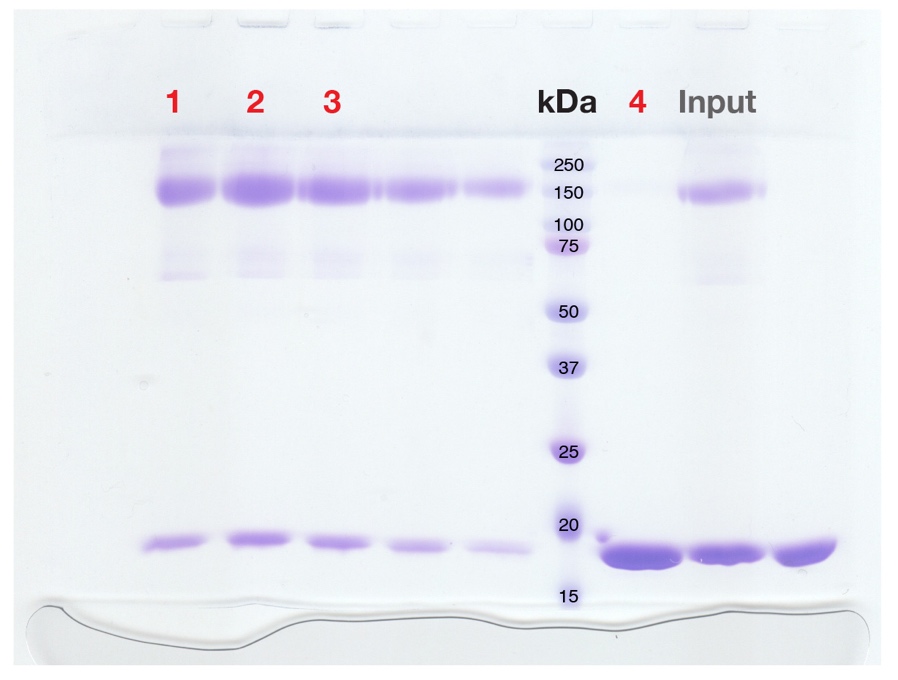
**

**Supplementary Figure S5.** Uncropped picture of the SDS-PAGE shown in Fig. 1 of the main manuscript. The lanes used for Fig. 1 are labeled accordingly. Molecular weights of the markers used are also indicated in kDa.

**Supplementary Table S1.** Data acquisition and processing information

| **Data collection and processing** | |  | **Model building and validation** | |
| --- | --- | --- | --- | --- |
| Microscope | Titan Krios G3 |  | Software | Phenix 1.20-4459 |
| Voltage (kV) | 300 |  | Model composition |  |
| Camera | Gatan Quantum-K3 |  | Non-hydrogen atoms | 8,477 |
| Magnification | 105,000 |  | Protein residues | 1,079 |
| Pixel size (Å) | 0.822 |  | Ligand residues | 4 |
| Energy filter | Gatan |  | MolProbity score ^a^ | 1.24 |
| Energy filter slit width (eV) | 20 |  | All-atom clashscore | 4.05 |
| Collection software | Serial EM |  | Rotamer |  |
| Defocus range (µm) | -0.8 to -1.8 |  | Favored (%) | 98.13 |
| Movies recorded | 20,512 |  | Outliers (%) | 0 |
| Frames per movie | 40 |  | Ramachandran |  |
| Camera dose rate (e^-^/px/s) | 15 to 20 |  | Favored (%) | 97.76 |
| Exposure rate (e^-^/Å^2^/frame) | 1.255 |  | Outliers (%) | 0 |
| Exposure time (s) | 1.491 |  | Z-score | 0.8 ± 0.26 |
| Cumulative exposure (e^-^/Å^2^) | 50.2 |  | R.m.s. deviations |  |
| Software used for processing | Relion 3.1.1 |  | Bond length (Å) | 0.006 |
|  |  |  | Bond angles (°) | 1.046 |
|  |  |  | Mean B-factors |  |
|  |  |  | Protein (Å^2^) | 59.98 |
| **Refinement** |  |  | Ligand (Å^2^) | 61.55 |
| Software | cryoSPARC v3.3.1 |  | CaBLAM outliers (%) | 2.81 |
| Initial particle images (no.) | 4,985,077 |  | Cβ outliers (%) | 0 |
| Final particle images (no.) | 552,926 |  | EM-Ringer score ^b^ | 2.76 |
| Symmetry | C_1_ |  | 0.5 FSC model resolution (Å) | 3.29 |
| 0.143 FSC half map (Å) | 3.18 |  | Map CC (mask) ^c^ | 0.81 |
| Map sharpening B-factor (Å^2^) | -149.5 |  | Map CC (peaks) ^c^ | 0.59 |
|  |  |  | Map CC (volume) ^c^ | 0.79 |

^a^ Model statistics were calculated using MolProbity ^5^.

^b^ Calculated based on local fit of side chains to map according to Barad et al. ^6^.

^d^ Real-space correlation coefficients of model-to-map fit as described in Afonine et al. ^7^.

**References**

1 Zivanov, J. *et al.* New tools for automated high-resolution cryo-EM structure determination in RELION-3. *Elife* **7**, e42166, doi:10.7554/eLife.42166 (2018).

2 Scheres, S. H. W. RELION: Implementation of a Bayesian approach to cryo-EM structure determination. *J. Struct. Biol.* **180**, 519-530, doi:10.1016/J.JSB.2012.09.006 (2012).

3 Punjani, A., Zhang, H. W. & Fleet, D. J. Non-uniform refinement: adaptive regularization improves single-particle cryo-EM reconstruction. *Nat. Methods* **17**, 1214-1221, doi:10.1038/s41592-020-00990-8 (2020).

4 Punjani, A., Rubinstein, J. L., Fleet, D. J. & Brubaker, M. A. cryoSPARC: algorithms for rapid unsupervised cryo-EM structure determination. *Nat. Methods* **14**, 290-296, doi:10.1038/Nmeth.4169 (2017).

5 Williams, C. J. *et al.* MolProbity: More and better reference data for improved all-atom structure validation. *Protein Sci.* **27**, 293-315, doi:10.1002/pro.3330 (2018).

6 Barad, B. A. *et al.* EMRinger: side chain-directed model and map validation for 3D cryo-electron microscopy. *Nat. Methods* **12**, 943-946, doi:10.1038/nmeth.3541 (2015).

7 Afonine, P. V. *et al.* Real-space refinement in PHENIX for cryo-EM and crystallography. *Acta Crystallogr. D Struct. Biol.* **74**, 531-544, doi:10.1107/S2059798318006551 (2018).
